# Spatiotemporal diversity and regulation of glycosaminoglycans in cell homeostasis and human disease

**DOI:** 10.1152/ajpcell.00085.2022

**Published:** 2022-03-16

**Authors:** Amrita Basu, Neil G. Patel, Elijah D. Nicholson, Ryan J. Weiss

**Affiliations:** ^1^Complex Carbohydrate Research Center, University of Georgia, Athens, Georgia; ^2^Department of Biochemistry and Molecular Biology, University of Georgia, Athens, Georgia

**Keywords:** chondroitin sulfate, glycosaminoglycans, heparan sulfate, regulation, transcription factors

## Abstract

Glycosaminoglycans (GAGs) are long, linear polysaccharides that are ubiquitously expressed on the cell surface and in the extracellular matrix of all animal cells. These complex carbohydrates play important roles in many cellular processes and have been implicated in many disease states, including cancer, inflammation, and genetic disorders. GAGs are among the most complex molecules in biology with enormous information content and extensive structural and functional heterogeneity. GAG biosynthesis is a nontemplate-driven process facilitated by a large group of biosynthetic enzymes that have been extensively characterized over the past few decades. Interestingly, the expression of the enzymes and the consequent structure and function of the polysaccharide chains can vary temporally and spatially during development and under certain pathophysiological conditions, suggesting their assembly is tightly regulated in cells. Due to their many key roles in cell homeostasis and disease, there is much interest in targeting the assembly and function of GAGs as a therapeutic approach. Recent advances in genomics and GAG analytical techniques have pushed the field and generated new perspectives on the regulation of mammalian glycosylation. This review highlights the spatiotemporal diversity of GAGs and the mechanisms guiding their assembly and function in human biology and disease.

## INTRODUCTION

The glycocalyx is a diverse network of complex carbohydrates that coats the surface of all animal cells. The major components of the glycocalyx, including glycolipids and glycoproteins, serve not only to protect the cell, but also function as the primary mediators of a cell’s interaction with its environment (1, 2). Glycosaminoglycans (GAGs) are long, linear polysaccharides and key members of the glycocalyx that play important roles in a variety of normal and pathophysiological cellular processes (3). These complex carbohydrates are composed of repeating disaccharide units containing amino sugar (e.g., glucosamine) and uronic acid (e.g., glucuronic acid) building blocks. These sugar moieties can be *O*- or *N*-sulfated at different positions, which give them a high overall negative charge. There are four major families of GAGs that are classified based on their disaccharide composition and biosynthesis: heparin/heparan sulfate (HP/HS), chondroitin/dermatan sulfate (CS/DS), keratan sulfate (KS), and hyaluronan (HA). In the interest of space, here we have focused on HS and CS/DS and their roles in cell homeostasis and human disease (4, 5). For further reading on the other GAG classes, we refer the reader to reviews by Karamanos et al. (6) and Caterson and Melrose (7).

**Figure 1.**
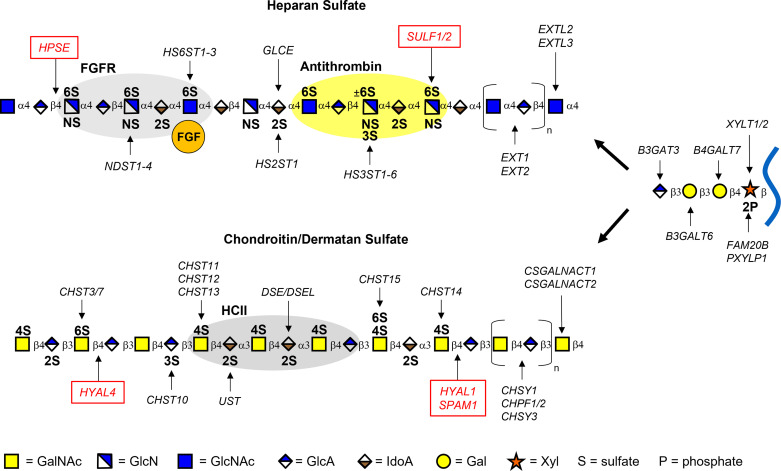
Heparan and chondroitin/dermatan sulfate biosynthesis. Assembly of the chains is performed by a large group of biosynthetic enzymes located in the Golgi. Heparan sulfate (HS) and chondroitin sulfate (CS)/dermatan sulfate (DS) share a common tetrasaccharide linker (Xyl-Gal-Gal-GlcA) that is attached to a serine residue of the core protein. HS and CS/DS assembly is initiated by EXTL3 (HS) or CSGALNACT1/2 (CS/DS), respectively, followed by polymerization and sulfation at specific sites. The gray and yellow ovals signify binding sites for ligands/proteins. FGF, fibroblast growth factor; FGFR, fibroblast growth factor receptor; HCII, heparin cofactor 2; GalNAc, *N*-acetylgalactosamine; GlcN, glucosamine; Gal, galactose; GlcA, glucuronic acid; GlcNAc, *N*-acetylglucosamine; IdoA, iduronic acid; Xyl, xylose.

HS and CS/DS are ubiquitously expressed in all animal cells and are attached to core proteins, known as proteoglycans, on the cell surface and extracellular matrix (ECM). These sulfated GAGs profoundly impact the biology of cells and tissues and act as structural components of connective tissue and the basement membrane (8). The biosynthesis of HS and CS/DS occurs in the endoplasmic reticulum (ER) and the Golgi and is catalyzed by a large family of biosynthetic enzymes (Fig. 1). As the chains are assembled, they undergo a series of modification reactions including sulfation and epimerization, which generates sulfated regions/domains that encode binding sites for ligands, such as growth factors and chemokines (9). Interestingly, various mammalian tissues and cell types exhibit distinct expression profiles for the biosynthetic enzymes and proteoglycans and, as a result, exhibit characteristic patterns of HS and CS/DS chain modifications. This structural heterogeneity is found temporally and spatially during development (10–12) and is altered when cells undergo transformation (13). The immense structural diversity and anionic nature of these polysaccharides enable them to interact with many protein ligands at the cell surface and ECM interface, thus impacting many facets of cell biology, including differentiation, cell signaling, angiogenesis, and coagulation (14). Despite the breadth of published studies detailing their biosynthesis in cells, the regulatory mechanisms that dictate tissue-specific patterns and resultant functions of HS and CS/DS remain poorly understood. Recent advances in genomics, chemical biology, and bioinformatics have led to novel insights into the regulation of GAG assembly in cells. In this review, we highlight GAG structure and function, biosynthesis, and their regulation in the context of cell biology and human disorders.

## GLYCOSAMINOGLYCAN STRUCTURE AND BIOSYNTHESIS

HS and CS/DS are linear polysaccharides that are assembled while attached to one of ∼77 known proteoglycan core proteins. These proteoglycans are differentially expressed in various tissues and cell types, and certain core proteins can also function as part-time proteoglycans, meaning they may exist with or without the GAG chains (15, 16). These heterogeneous polysaccharides are biosynthesized in a nontemplate-driven manner by a family of enzymes encoded by nearly 40 genes (17). The process of HS and CS/DS biosynthesis begins in the cytoplasm with the synthesis of 5-uridine-diphosphate (UDP) sugars that are then transported to the Golgi apparatus (18). The assembly of both HS and CS/DS is initiated by the formation of a common tetrasaccharide structure catalyzed by xylosyltransferase I/II (XYLT1/XYLT2), B4GALT7, B3GALT6, and B3GAT3. This linker region contains xylose (Xyl), two galactose (Gal) molecules, and d-glucuronic acid (GlcA), with xylose covalently bound to the serine residue of the proteoglycan core protein (Fig. 1). The xylose sugar can be phosphorylated or dephosphorylated at the C2 hydroxyl group by FAM20B and PXYLP1, respectively, which regulates the rate of formation of the linker region and subsequent chain elongation (19–21). HS synthesis is initiated once a *N*-acetyl-d-glucosamine (GlcNAc) residue is added by EXTL3 (22, 23), and CS/DS assembly begins upon addition of *N*-acetyl-d-galactosamine (GalNAc) by CSGALNACT1 or CSGALNACT2 (24, 25). The *N*-acetylhexosaminyltransferase, EXTL2, can transfer GalNAc and GlcNAc to the common GAG linkage region (26), although the exact function of this enzyme in GAG assembly remains unclear (27). Interestingly, this enzyme has been shown to specifically control HS biosynthesis via addition of a phosphorylated GlcNAc residue to the tetrasaccharide linkage, which thereby terminates chain elongation (28). HS polymerization occurs through the addition of alternating GlcNAc and GlcA residues by the heterodimeric co-polymerase complex containing exostosin-1 (EXT1) and exostosin-2 (EXT2) (29, 30). CS polysaccharides consist of alternating units of *N*-acetyl-d-galactosamine (GalNAc) and GlcA and are synthesized by a series of seemingly redundant glycosyl- and sulfotransferases. DS is structurally related to CS; however, DS chains uniquely contain GalNAc and d-iduronic acid (IdoA) repeating disaccharide units. CS/DS elongation occurs upon addition of repeating GalNAc and GlcA repeating units by any combination of a hetero-oligomer complex of chondroitin sulfate synthase 1 (CHSY1), chondroitin polymerizing factor 1/2 (CHPF1/CHPF2), and chondroitin sulfate synthase 3 (CHSY3), depending on tissue-specific expression of the enzymes (31–33).

After elongation, HS and CS/DS chains are sulfated at specific sites by multiple sulfotransferase enzymes, which creates highly sulfated domains that can interact with protein binding partners (9, 34). HS sulfation is thought to proceed in a processive manner driven by enzyme substrate specificities. First, GlcNAc residues are *N*-deacetylated and subsequently *N*-sulfated by *N*-deacetylase/*N*-sulfotransferases (NDST1-4), followed by addition of a 2-*O*-sulfate group to C2 of GlcA or IdoA (HS2ST1), which is generated by the C5-epimerase GLCE, and subsequent 6-*O*-sulfation of C6 on GlcN/GlcNAc residues (HS6ST1-3) (35). In addition, relatively rare 3-*O* sulfate groups can be added to the C3 position of specific *N*-sulfoglucosamine residues by seven 3-*O* sulfotransferases (HS3ST1–6) (36). CS chains can be *O*-sulfated at the C4 (CHST11-14) and C6 (CHST3/7/15) of GalNAc residues and at C2 (UST) and more rarely at C3 (CHST10) of GlcA (37). In addition, GlcA residues may be epimerized into IdoA residues via DSE/DSEL and 2-*O* sulfated by UST, resulting in structurally related chondroitin/dermatan sulfate (CS/DS) hybrid chains (38) (Fig. 1). Overall, the variation in polymer length and sulfation patterns of HS and CS/DS regulate their interactions with GAG-binding proteins and ligands in the ECM. Since sulfation is not uniform across the polysaccharide chain and most modification reactions do not go to completion, GAG chains are variably sulfated at structurally diverse, containing both highly sulfated regions and regions lacking all or most of these modifications (39–41).

After assembly and presentation on the cell surface and ECM, both HS and CS chains can be further processed by extracellular sulfatase and/or glycosidase enzymes. These secreted factors are implicated in remodeling of the ECM and regulating GAG-protein interactions via tuning GAG assembly in the extracellular space. A classic example is heparanase-1 (HPSE), which is an endo-β-d-glucuronidase encoded by the gene *HPSE*. This enzyme degrades HS chains through cleavage of the β-1,4 glycosidic bond between GlcA and GlcN at sites of low sulfation (NA domains) (42). HS degradation by HPSE results in the release of 5–10 kDa fragments of HS, which facilitates structural alterations to the ECM and basement membrane and is associated with disease states, such as tumor metastasis and sepsis (43). In fact, it has been shown that degradation of HS chains by HPSE enhances the accessibility of the proteoglycan core protein to enzymatic cleavage by matrix metalloproteases and other proteases (44). Heparanase-2 (HPSE2), a secreted protein that shares 40% homology to HPSE, interestingly does not exhibit glycosidase activity but can block HPSE activity and act as a tumor suppressor (45).

The endo-6-*O*-sulfatases, SULF1 and SULF2, are secreted proteins that regulate the binding and activity of growth factors through removal of a subset of 6-*O* sulfate groups from highly sulfated regions (NS domains) of HS (46, 47). Consequently, cell signaling can be attenuated (fibroblast growth factors) or promoted (canonical WNT) by SULF-mediated HS desulfation (48). For example, studies have implicated the SULFs acting as both a tumor suppressor (49) and promoter of tumorigenesis (50–52) depending on cancer type. In vitro and in vivo models have revealed that SULF1 and SULF2 are functionally redundant in certain contexts, such as during skeletal development (53). Interestingly, the isoforms display distinct expression patterns among various adult tissues (54) and brain regions (55) yet exhibit overlapping expression patterns during embryonic development (53). Together, this suggests that SULF1 and SULF2 dynamically influence HS-protein interactions by regulating tissue-specific HS fine structure. In contrast, CS endosulfatases have not been discovered (56), yet there is some evidence that the lysosomal exosulfatase, arylsulfatase B (ARSB), can localize at the cell membrane and impact CS levels under certain pathophysiological conditions (57, 58). Three mammalian hydrolases capable of cleaving CS chains have been identified, including PH20 (SPAM1), hyaluronidase 1 (HYAL1), and hyaluronidase 4 (HYAL4). Although all three can also cleave hyaluronic acid glycosaminoglycans, HYAL4 is predominantly an endo-β-*N*-acetylgalactosaminidase that degrades CS motifs containing 2-*O* and 6-*O* sulfated subunits (CS-D). HYAL4 has been shown to cleave CS chains on multiple proteoglycans, including serglycin, aggrecan, and CD44, and its expression is altered in certain disease states, such as cancer (59). Altogether, the activity and expression of biosynthetic enzymes and secreted remodeling factors discussed here have a significant impact on the huge chemical diversity of HS and CS/DS chains and their resultant functions across tissue/cell types and disease states.

## SPATIAL AND TEMPORAL DIVERSITY OF GLYCOSAMINOGLYCANS

**Figure 2.**
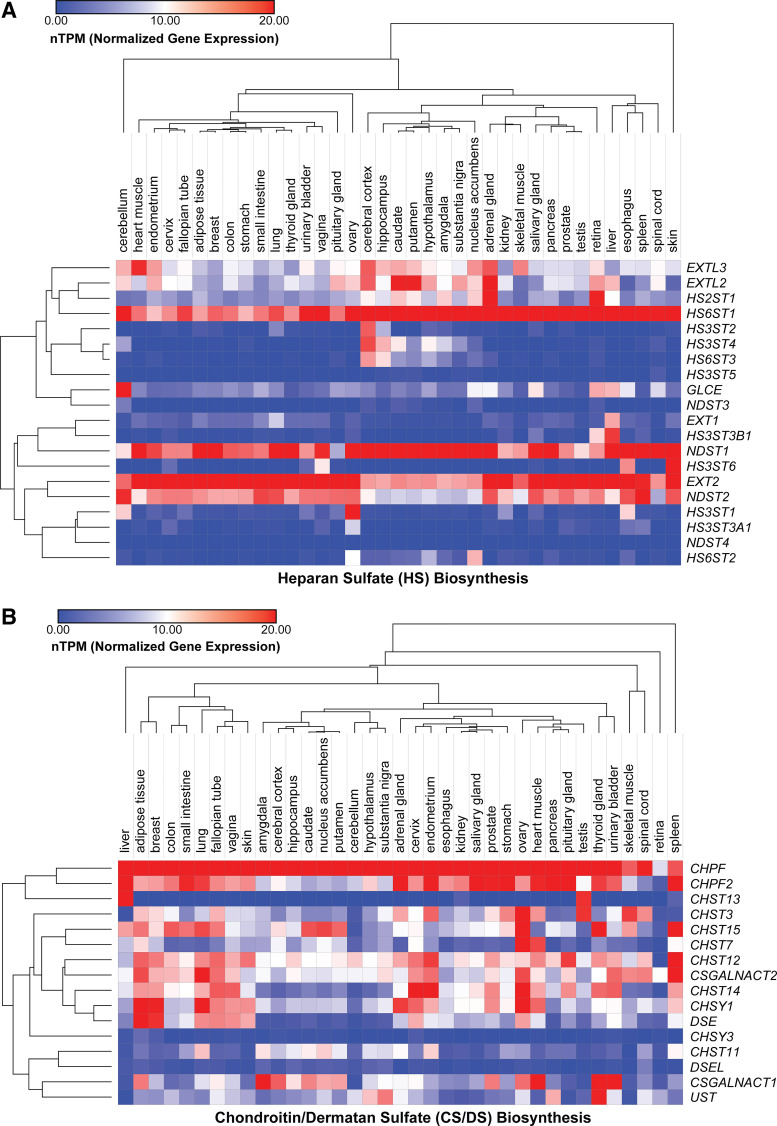
Expression profiles of glycosaminoglycan (GAG) biosynthetic enzymes in human tissues. Heatmaps show transcript expression levels for heparan sulfate (HS, *A*) and chondroitin sulfate/dermatan sulfate (CS/DS, *B*) biosynthetic enzymes in 37 human tissues based on RNA sequencing (normalized transcripts per million, nTPM). Hierarchical clustering was performed based on the Pearson correlation. Data were obtained from GTEx and is based on The Human Protein Atlas version 21.0 and Ensembl version 103.38.

The structural diversity of GAG polysaccharides is vast among different species and can vary temporally during development and spatially in tissues. Consequently, GAGs contribute significantly to cell- and organ-specificity, cell lineage (60–62), tissue/organ function (63–65), and pathophysiological conditions, such as tumorigenesis (66) and inflammation (67). The development of pattern-specific antibodies for CS (68) and HS (69, 70) facilitated the initial discovery of cellular and tissue distribution of GAGs during development. Recent advances in next generation sequencing and state-of-the-art analytical tools have enabled detailed profiling of the spatiotemporal expression of GAG enzymes (10, 71) and their diverse structure/composition (72) across various organisms, cells, and tissues. In Fig. 2, we present tissue-specific gene expression of all the enzymes involved in HS and CS biosynthesis across 37 human tissues obtained from the Human Protein Atlas (73, 74). This data emphasizes differential gene expression of individual HS and CS biosynthetic enzymes across various human tissues and highlights distinct clustering of certain enzymes based on similar expression signatures. Generally, genes encoding for enzymes involved in elongation of the GAG chains, such as *EXT2* and *CHPF1/2*, are highly expressed across most tissues, whereas sulfotransferase enzymes, such as HS3STs and HS6STs, tend to cluster with localized expression patterns, especially in regions of the brain (75). Clearly, there are dominant isoforms across all tissues for a given enzyme family (*NDST1, HS6ST1, EXT2,* and *SULF2*), and certain genes have low expression across all 37 tissues (e.g., *NDST3* and *CHSY3*). Overall, CS genes are more highly expressed, whereas HS genes show more tissue specificity, depending on the enzyme subclass. Although it is obvious that GAG expression is regulated at the transcriptional level, the mechanisms controlling this spatiotemporal regulation in cells and tissues remain still unclear.

Many studies up to this point have utilized the availability of animal models to explore the temporal and spatial diversity of GAG assembly. For example, it has been shown that total HS amounts vary widely when isolated from different mouse tissues (76). Along with changes in overall amounts of HS, their composition and sulfation patterns are diverse across different cell types, allowing for different capacities to bind ligands and receptors involved in cell signaling (54). Furthermore, multiple diverse forms of CS/DS (CS A–E) with distinct sulfated disaccharide repeats are present across various species and tissues. Depending on sulfation pattern and IdoA content, CS/DS chains can be classified into different subtypes: CS-A [GlcA-GalNAc(4S)], CS-B/DS [IdoA(2S)-GalNAc(4S)], CS-C [GlcA-GalNAc(6S)], CS-D [GlcA(2S)-GalNAc(6S)], CS-E [GlcA-GalNAc(4S,6S)], and CS-iE [IdoA-GalNAc(4S,6S)] (77, 78). Interestingly, these different types of CS exhibit distinct spatiotemporal expression across tissues, particularly in the brain (79, 80). In the human brain, CS is highly expressed and experiences a significant shift from CS-C to CS-A during development (81). In addition, CS gene expression and composition is altered in the postnatal mouse cerebellum during development, with a significant shift from CS-E to CS-D/DS (82). Finally, HS and CS composition were also found to differ significantly between the model organisms *Hydra vulgaris*, *Drosophila melanogaster*, and *Caenorhabditis elegans*, which represents a potential for evolutionary distinct functional motifs across metazoans (83).

Recently, publicly available RNA sequencing data from various mouse tissues and across developmental stages were comprehensively analyzed and integrated to profile the spatial, temporal expression for genes involved in HS biosynthesis (84). Similar to the human expression data (Fig. 2), many of the enzymes involved in HS sulfation in mice show distinct spatial expression patterns, particularly among different isoforms. Within the family of NDST enzymes, for example, *Ndst1* and *Ndst2* expression were detected at significant levels across all 21 mouse tissue types, whereas *Ndst3* and *Ndst4* were both only detected in the cerebral cortex and testis. In the same study, temporal expression of certain enzymes was shown to vary significantly across different time points from embryonic to postnatal development in the mouse forebrain. For instance, certain 3-*O* sulfotransferases (*Hs3st1* and *Hs3st5*) steadily increase in expression during embryonic development but are attenuated at the postnatal stage. Moreover, *Sulf1* expression decreases throughout the embryonic stage and maintains low expression throughout the postnatal stage, whereas *Sulf2* exhibits a general decrease in expression throughout the embryonic stage but fluctuates with no discernible pattern of expression in the postnatal stage. Other studies have revealed significant alterations in GAG composition during stem cell differentiation and fate commitment (85). Interestingly, ∼80% of GAGs produced by undifferentiated mouse embryonic stem cells (mESCs) contain a low-sulfated form of HS (with low overall CS levels), but upon transition to committed cell types, their HS chains become increasingly sulfated with a concurrent increase in CS synthesis (86, 87). Comprehensive profiling of the glycome in human embryonic stem cells (hESCs) and human induced pluripotent stem cells (hiPSCs) also revealed overall low sulfation of HS and predominantly 4-mono-sulfated CS disaccharides in both populations, and total amounts of these “immature” GAGs were significantly higher when compared with other nonstem human cell lines (62). Differentiation of mESCs along the mesodermal lineage to the hemangioblast stage results in transient expression of a unique 3-O sulfated HS epitope that is a marker for distinct cell populations along the lineage (88). These transient changes in GAG expression during differentiation presumably impacts GAG-growth factor interactions and downstream cell signaling pathways. The mechanisms that control these changes in GAG composition during different developmental stages are still unknown. Presumably, epigenetic factors (e.g., chromatin remodeling complexes), which play key well-studied roles in mammalian development (89), may also tune GAG gene expression as a way to respond to extracellular cues and impact stem cell differentiation and pluripotency.

## REGULATORY MECHANISMS OF GLYCOSAMINOGLYCAN ASSEMBLY

**Figure 3.**
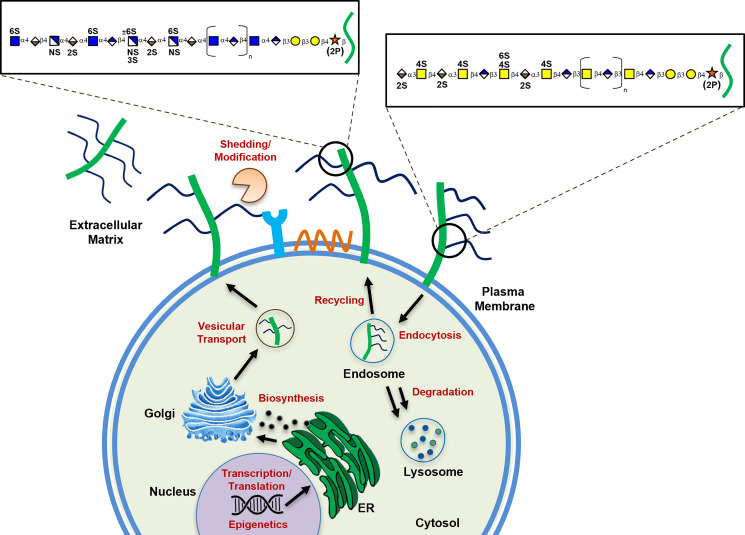
Regulation of glycosaminoglycan (GAG) biosynthesis. Illustration depicting regulatory pathways controlling heparan sulfate (HS) and chondroitin sulfate (CS)/dermatan sulfate (DS) biosynthesis in the cell. Red lettering indicates potential steps of regulation before presentation of GAG chains in the extracellular space.

Over the past few decades, GAG biosynthetic enzymes have been identified, cloned, biochemically characterized, and mutated in cells and model organisms (17). Although much is known about the enzymes and their impact on HS/CS composition, little is known about the mechanisms that dictate spatiotemporal patterning of the chains and trigger their dysregulation in human disease (5, 90, 91). The organization of sulfated residues along the chains, in part, reflects the specificity of the enzymes (9); however, other nonenzymatic factors could regulate patterning of the chains and expression of the biosynthetic enzymes and proteoglycan core proteins. Before HS/CS chains are presented as proteoglycans on the cell surface and in the ECM, it is conceivable that many steps along the pathway could be regulated and thus impact GAG structure/function in the extracellular space, including: *1*) the expression of the core proteins or biosynthetic enzymes by transcriptional, translational, or epigenetic factors, *2*) the organization and activity of enzymes in the ER and Golgi, *3*) nucleotide sugar and 3′-phosphoadenosine-5′-phosphosulfate (PAPS) levels, *4*) chaperone and/or scaffolding proteins in the ER and Golgi during biosynthesis, *5*) rate of turnover and secretion, and *6*) other unknown factors (Fig. 3). Here, we will discuss potential regulatory mechanisms and highlight studies that have shed light on the regulation of GAG assembly.

### Regulation of Enzyme Expression and Activity

As aforementioned, the expression of GAG biosynthetic enzymes and the proteoglycan core proteins varies across different tissues and is tightly regulated during development. Early studies described the impact of growth factors on the regulation of proteoglycan expression and GAG chain length. The family of fibroblast growth factors (FGFs) was shown to activate expression of the proteoglycan, syndecan-1 (Sdc1) (92, 93). In a related study, the activation of Sdc1 expression in mouse keratinocytes and fibroblasts was growth factor-dependent and initiated by transcription factors and response elements upstream of Sdc1 (94). In addition, treatment of arterial smooth muscle cells with platelet-derived growth factor (PDGF) led to increases in CS/DS chain length, whereas transforming growth factor-β1 (TGFβ1) treatment resulted in enhanced mRNA levels of biglycan, a CS proteoglycan (CSPG) (95). TGFβ1 also was shown to augment CS biosynthesis by upregulating CHSY1 expression via the MAPK signaling pathway (96). Another report showed a role for TGFβ1 in regulating CS/DS chain polymerization (97).

Other genetic factors, such as 5′ untranslated region (UTR) and 3′ UTR mRNA sequences (98–100), microRNAs (101–103), and epigenetic factors (104–106) have been shown to affect expression of the enzymes and core proteins, which in turn affects the assembly of the chains, their binding properties, and their downstream biological activity. Multiple transcriptional regulatory elements have been reported to play a role in controlling GAG assembly and core protein expression (107–119). For example, an astrocyte-specific basic leucine zipper transcription factor, *OASIS*, was recently shown to positively regulate CHST3 expression during spinal cord injury (120). In addition, alternative splice forms of certain enzymes can be expressed in a spatiotemporal manner and impact biosynthesis. For example, a shorter alternative splice form of *NDST1* (“NDST1B”) is expressed at a low level in normal tissue but is overexpressed in certain cancers, where it might function in a dominant negative manner by replacing active endogenous NDST1 in the HS enzyme complexes during biosynthesis (121).

Recent advances in genomic technologies and bioinformatic tools have enhanced our ability to explore the genome to identify novel regulatory factors involved in GAG assembly. A recent data mining effort utilizing publicly available chromatin immunoprecipitation sequencing (ChIP-Seq) and RNA sequencing data uncovered the transcription factor, MEF2C, as a potential regulator of HS and CS enzyme expression (122). Bioinformatic analyses examining the promoter region of all HS enzymes for enrichment of transcription factor binding motifs led to the discovery of ZNF263 as a novel transcriptional repressor of *HS3ST1* and *HS3ST3A1* expression (123). The advent of CRISPR/Cas9 as a genetic tool (124, 125) and associated screening technologies (126, 127) has enabled unbiased genome-wide searches for nonenzymatic factors regulating GAG assembly. Our group recently reported the development of a genome-wide CRISPR screen to uncover novel factors regulating HS assembly in human cells. This genetic screen uncovered many potential regulatory pathways for HS biosynthesis and, importantly, revealed that the histone demethylase, KDM2B, controls HS-protein interactions via epigenetic regulation of *SULF1* expression (128). Another CRISPR screen designed to identify host factors of Sindbis virus infection found that mutating *COG4*, a member of the conserved Golgi complex, results in a defect in HS biosynthesis in human colorectal carcinoma cells (129).

### Enzyme Localization and Golgi Homeostasis

The Golgi can be divided into cis, medial, and trans compartments. Interestingly, the majority of HS enzymes localize to the cis/medial stacks, whereas CS enzymes are located in the trans-Golgi network (130, 131). Disruption of the Golgi network with the inhibitor brefeldin-A results in mis-localization of multiple CS enzymes and defects in CS polymerization (132). In addition, deletion of the stem region of HS6ST isoforms inhibits their localization to the Golgi and reduces sulfotransferase activity (133). β-Secretase was also found to indirectly regulate 6-*O* sulfation of HS through truncation of HS6ST3 thus altering its cellular localization (134). Moreover, knockout mice with mutations in the Golgi-resident bisphosphate nucleotidase 2 (BPNT2), which catalyzes the breakdown of 3′-phosphoadenosine-5′-phosphate in the Golgi, led to impaired 4-*O* sulfation of CS (135). A genome-wide screen recently revealed that the transmembrane protein, TM9SF2, is a novel host factor for chikungunya virus infection and a critical regulator of *N*-sulfation of HS and localization and stability of NDST1 in the Golgi (136). Also, the Golgi-resident transmembrane protein 165 (TMEM165) was discovered to be a manganese transporter and crucial regulator of GAG biosynthesis. Knockout of TMEM165 resulted in a defect in Mn^2+^ availability in the Golgi and a profound decrease in HS/CS chain elongation. Supplementation with Mn^2+^ rescued the GAG polymerization process, suggesting that the availability of metal ion cofactors is also important for GAG synthesis in the Golgi (137). Other factors, such as the Drosophila GOLPH3 homolog, have been shown to regulate the retrograde trafficking of EXT1 and EXT2 (138).

### The “GAGosome”

The “GAGosome” hypothesis was first proposed by Esko and Selleck (139) suggesting that a physical complex of the biosynthetic enzymes exists in the Golgi. This theory was built upon early evidence suggesting that rapid biosynthesis of the chains is due to the cooperative activity of the enzymes (140, 141). Another early report showed evidence of specific interactions between XYLT1 and B4GALT7 during CS biosynthesis (142). Multiple studies since then have provided additional evidence to support this concept. The first example is the seminal discovery that heterooligomeric complexes form to polymerize both HS (EXT1/EXT2) (30) and CS chains (CHSY1/CHSY3/CHPF1/CHPF2) (31). In addition, the HS C5-epimerase GLCE was also shown to directly interact with the 2-*O* sulfotransferase HS2ST1 in vivo, which impacts epimerase stability and translocation to the Golgi (143). More recently, immunoprecipitation experiments showed that EXT1 and EXT2 can directly interact with NDST1, which affects HS sulfation levels and *NDST1* expression (144). Further studies and new methods are needed to elucidate other nonenzymatic factors, such as chaperones and/or scaffolding proteins that may regulate enzyme activity and impact GAG synthesis in the Golgi.

### Proteoglycan Structure and HS/CS Attachment Sites

In certain contexts, the proteoglycan core protein may compete with the biosynthetic enzymes and may be the limiting step in GAG biosynthesis. Certain protein domains of proteoglycans have been shown to influence CS or HS utilization of their GAG attachment sites and sulfation patterns. In an earlier study, the globular domain of glypican-1 (GPC1), a major glycosylphosphatidylinositol-anchored cell surface HS proteoglycan, was found to be a key structural motif that influences whether HS or CS chains are added to the core protein. Removal of this domain resulted in a shift from ∼90% HS to ∼90% CS chains on the protein (145). Another study investigated whether the organization of non-GAG bearing domains from two matrix proteoglycans, perlecan and aggrecan, can impact HS/CS occupancy. Intriguingly, expression of perlecan/aggrecan chimeras with different non-GAG bearing domains resulted in significant changes to the HS/CS ratio on the core proteins, depending on which domains were present. This suggested that utilization of attachment sites for HS and CS may be influenced, at least in part, by non-GAG-bearing domains (146). Kokenyesi and Bernfield discovered that mouse Sdc1 contains five GAG attachment sites containing either HS or CS chains, depending on the protein domain. HS was found solely at the N-terminus while CS chains were attached to the C-terminus sites, suggesting that core protein structure regulates HS/CS occupancy (147). A more recent study compared the sulfation levels of HS attached to mouse syndecan-2 (Sdc2) and syndecan-4 (Sdc4). Interestingly, they found that insertion of the N-terminal domain sequence of Sdc2 into Sdc4 drove a significant increase in 6-*O* sulfation of its HS chains compared with the native Sdc4 sequence. These results provide further evidence of the existence of core protein-determined HS sulfation patterns (148). Newer analytical approaches have helped address these questions of site-specific glycosylation by identifying HS/CS attachment sites of proteoglycans (15, 149, 150).

The activity of certain enzymes can regulate HS/CS assembly and act as a quality control mechanism. As discussed earlier, transient phosphorylation of the C2 position of xylose in the linker region functions as a molecular switch to regulate GAG synthesis. In fact, loss of FAM20B-dependent phosphorylation leads to incomplete linkage regions that are capped with sialic acid, thus halting elongation (151). Interestingly, sulfation of the linkage region at various positions can function as a regulatory signal in chain initiation. For instance, 6-*O* sulfation of the two galactoses in the linkage region influences B3GAT3 (152) and B4GALT7 activity (20), whereas 4- or 6-*O* sulfation can significantly enhance *N*-acetylgalactosaminyltransferase-1 (CSGALNACT1) activity (25). In addition, sulfation of the GlcA residue in the tetrasaccharide linker by HNK-1 (CHST10) can suppress CS assembly in certain core proteins (153). EXTL2 can function as both a terminator and activator of GAG synthesis through addition of GlcNAc and GalNAc to the linker region. Addition of α-GalNAc to the linker region terminates CS biosynthesis, whereas adding GlcNAc initiates HS biosynthesis (26). Suppression of EXTL2 and EXTL3 leads to reduced HS biosynthesis (154), yet knockdown of EXTL3 also results in increased chain length, indicating a complex regulatory mechanism (23). The HS enzyme NDST2 exhibits high *N*-deacetylase but weaker *N*-sulfotransferase activity compared with NDST1 (155), yet a recent study showed that this enzyme could also stimulate HS chain elongation through an unknown mechanism (156). Intriguingly, the addition of a 4-*O* sulfate group to the nonreducing terminal *N*-acetylgalactosamine during CS synthesis by CHST11 was found to facilitate chain elongation by CHSY1/CHPF in cooperation with the *N*-acetylgalactosaminyltransferase, CSGALNACT2 (157). Overall, these findings have exposed novel mechanisms dictating CS or HS chain attachment and elongation at certain serine residues of proteoglycans; however, there is still much unknown on this topic.

## GLYCOSAMINOGLYCAN REGULATION IN HUMAN DISEASE

Dysregulation of GAG biosynthesis has been implicated in a variety of human disorders, and alterations in GAG structure have a direct impact on their function in disease states. Such defects can occur within the biosynthetic machinery, altering the polymerization or modifications of the chains, which affects their ability to bind proteins, such as growth factors and chemokines, to impact tissue function and physiology. Depending on the underlying alterations in GAG assembly, diverse pathophysiological phenotypes have been observed within a given disorder (e.g., congenital disorders). Here, we have outlined how the dysregulation of GAG assembly impacts human disease.

### Genetic Disorders

**Figure 4.**
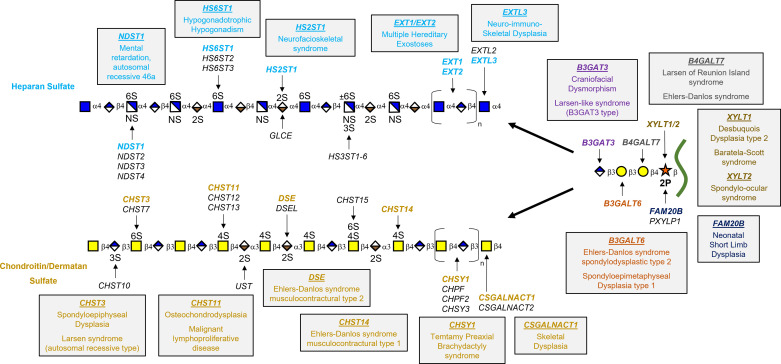
Congenital disorders of glycosaminoglycan biosynthesis. Diagram highlighting genetic disorders caused by mutations in key biosynthetic enzymes for heparan sulfate (HS) (blue text) and chondroitin sulfate (CS)/dermatan sulfate (DS) (gold text) assembly and the shared tetrasaccharide linker region. A complete reference list is available in Ref. 158.

A variety of mutations in genes encoding GAG biosynthesis enzymes have been implicated in several relatively rare human diseases (Fig. 4). Interestingly, defects in GAG biosynthesis usually portray clinical features in bone or cartilage, ligaments, or subepithelial layers (158). For example, dysregulation of HS chain polymerization is a key feature in multiple hereditary exostoses (MHE), an autosomal dominant skeletal disorder hallmarked by the formation of benign cartilage-capped tumors. This disorder is caused by a heterozygous loss-of-function mutation in *EXT1* or *EXT2* (159, 160). HS synthesized by chondrocytes at the growth plate of long bones is crucial for growth factor signaling. Mutations altering HS chain polymerization leads to truncation of the HS chains and a consequential decrease in HS levels, which results in disruption of signaling pathways [e.g., FGF, bone morphogenetic protein (BMP), and Hedgehog], leading to exostoses formation (161, 162).

A variety of mutations in genes involved in GAG biosynthesis have been shown to cause Ehlers–Danlos syndromes (EDS), a heterogeneous group of hereditary connective tissue disorders that are characterized by hypermobility of joints, hyperextensible skin, and weakened or fragile connective tissue (163). One variant of the disease, EDS musculocontractural type 2, is caused by homozygous mutations in *DSE*, which impedes DS formation via a reduction in GlcA epimerization to IdoA (164). In addition, mutations in *CHST14*, which encodes for dermatan 4-*O*-sulfotransferase 1 (D4ST1), results in the absence of DS and excess CS synthesis, causing EDS musculocontractural type 1 (165). We have highlighted multiple human disorders caused by genetic mutations in GAG enzymes in Fig. 4. Currently, there are limited therapies for these patients, therefore understanding the regulation of the genes and proteins involved in GAG biosynthesis will help identify targets for new treatment strategies.

### Neurodegenerative Diseases

Many neurological disorders can be traced to the dysregulation of GAGs, including tauopathies (166–168), Parkinson’s disease (169), multiple sclerosis (170), and neuroinflammation during spinal cord injury (171). HS has been repeatedly shown to play a key role in forming protein aggregates in various neurodegenerative disorders (172). It was originally reported that highly sulfated GAGs such as heparin, a highly sulfated form of HS produced in mast cells, promote aggregations of tau by competing with microtubules as binding sites (173). Heparin binding of tau induces a conformational change that consequently exposes previously masked phosphorylation sites. Different protein kinases can phosphorylate these newly exposed sites and lead to tau hyperphosphorylation, which is a hallmark of a variety of tauopathies, including Alzheimer’s disease, Pick’s disease, and progressive supranuclear palsy (174, 175). Recently, a rare 3-*O*-sulfation modification of HS was implicated in the spread of tau due to this modification’s enhancement of tau-HS binding (176). Furthermore, 6-*O*-sulfation of HS was also revealed to bind to and compete with cellular intake of tau (177). These findings present potential new therapeutic avenues to treat Alzheimer’s progression.

HS has also been suggested to play a role in forming protein aggregates of α-synuclein in Parkinson’s disease. Different sulfation patterns of HS allow for N-terminal domain interactions of the protein to promote the formation of α-synuclein fibrils and promotes their aggregation. On the other hand, the role of HS in multiple sclerosis (MS) etiology and pathology is less understood. Multiple studies have highlighted the beneficial and deleterious effects of the HS degrading enzyme, HPSE, in MS. In a mouse model of experimental autoimmune encephalitis (EAE), inhibition of HPSE resulted in reduced EAE (178). It is thought that increased expression of HPSE in the central nervous system (CNS) of EAE mice promotes MS disease progression and T cell invasiveness. Other limited studies have suggested that HPSE plays a beneficial role in reducing inflammation and blocking T cell migration into the central nervous system during MS pathogenesis (179). Future work will need to fully elucidate the effects of HPSE expression and activity on MS progression. Contrary to HS, CSPGs have been highly implicated in MS progression and neuroinflammation during spinal cord injury. CS is the main component of “glial scars” that form after spinal cord injury and act as inhibitors of axon regrowth (180). Therefore, there is much interest in targeting CSPGs during spinal cord injury (181). CSPGs are upregulated in plaques in patients with MS, particularly in white matter (182) and play a proinflammatory role during MS progression (183, 184).

### Inflammation

The varying sulfation patterns of HS allow for binding interactions with a wide array of ligands and receptors. Chemokines are a family of chemoattractant signaling proteins known to bind to HS across sulfated domains and are involved in inflammatory and immune responses via the recruitment of leukocytes (185). This binding results in a phenomenon called chemotaxis in which a concentration gradient of immobilized chemokines is established at the cell surface. Almost all chemokines have been shown to bind HS/heparin, and this process is thought to be the mechanism by which circulating blood leukocytes roll to positions along the endothelium to sites of inflammation (186).

Heparin is one of the most widely prescribed drugs in the world. Its potent anticoagulant activity makes it the drug of choice for treating deep vein thrombosis and pulmonary embolism in the acute care setting. The molecular basis of the anticoagulant action of heparin is linked to its ability to bind to the serine protease inhibitor antithrombin, leading to inhibition of the coagulation cascade (187). Of the 12 million patients treated with heparin in the USA annually, up to 5% of patients can develop a life-threatening autoimmune response triggered by the interaction between heparin and a platelet-derived chemokine, platelet factor 4 (PF4), known as heparin-induced thrombocytopenia (HIT). Heparin binds to PF4 and forms a supramolecular complex that is recognized by IgG antibodies, which results in promoting thrombin generation and other complications (188). Interestingly, prior studies have shown that PF4 binding to heparin depends on distinct sulfated sites compared with AT, suggesting that a safer form of anticoagulant heparin could be developed (189, 190).

### Cancer

**Figure 5.**
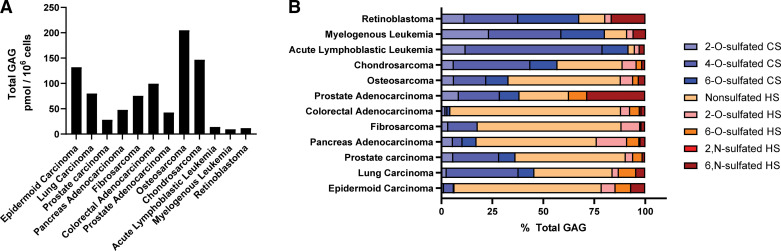
Glycosaminoglycan (GAG) profiling across various human cancer cell lines. Total heparan sulfate (HS)/chondroitin sulfate (CS) amounts (*A*) and disaccharide composition (*B*) vary between human cancer cells, as determined by RP-HPLC (Data obtained from Ref. 192). Sulfation patterns are grouped for CS (blue colors) and HS (red/orange colors).

Structural heterogeneity of the GAG chains also varies when cells undergo transformation (191). Alterations in GAG structure and function have been implicated in cancer growth, progression, and tumor metastasis (37). Cancer cells can dynamically regulate the structure and sequence of cell-surface and extracellular matrix HS, and many cancer cells secrete proangiogenic factors (e.g., vascular endothelial growth factors) that bind to HS on the cell surface and stimulate tumor angiogenesis and metastasis (13). Certain cancers display an increase in overall sulfation levels causing defects in growth factor signaling pathways that contribute to tumorigenesis. In fact, a recent study profiled HS/CS across various human cancer lines by reverse-phase HPLC (192). This study revealed immense structural diversity and differences in total amount of HS/CS, depending on the tissue of origin (see Fig. 5). FGF2 is a growth factor that binds to such sites and can increase cell proliferation in various cancers (193, 194). Since HS can promote cell-cell and cell-ECM adhesion (195, 196), many cancers show reduced HS levels (197), thereby promoting metastasis. The secreted 6-*O*-endosulfatase, SULF1, is downregulated in breast cancer, which results in high basal 6-*O*-sulfation levels and increased cell migration and invasion (198). HS has also been shown to play a role in epithelial to mesenchymal transition by binding growth factors in the tumor microenvironment (199). In the same way, CS has been associated with almost all the hallmarks of cancer, including proliferation, migration, invasion, angiogenesis, and metastasis (200). Many cancers show CS-E overexpression (201, 202), which allows for increased binding of vascular endothelial growth factor (VEGF) in ovarian adenocarcinomas (203), negative regulation of Wnt/β-catenin signaling in breast cancer (204), and increased metastasis of Lewis Lung carcinoma (201). Increased prevalence of 6-*O*-sulfation and nonsulfated disaccharides of CS are also implicated in the malignant phenotype of pancreatic cancer (205).

## CONCLUSIONS AND PERSPECTIVE

GAGs play many roles in essential cellular processes, including cell proliferation, signaling, and development, and interact with a diverse set of proteins and ligands in the extracellular space. Their distinct functions in biology are directly linked to their inherent structural diversity. As detailed above, there is ample evidence that GAG biosynthesis is spatiotemporally regulated in cells. Advances in genomics have enabled genome-wide profiling of GAG expression across tissues, and the development of effective analytical tools has supported the detailed structural analysis and distribution of GAG structures across cell types. Although a significant amount of research has been done to understand GAG structure and function, further investigation is needed to elucidate the distinct regulatory mechanisms controlling GAG assembly. One of the current challenges in the field is the analysis of intact GAG chains and longer oligosaccharides at high resolution. Classically, GAG structural information has been obtained via profiling the composition of the chains after enzymatic/chemical depolymerization of the chains (83, 149, 206) or sequencing intact purified oligosaccharides (“top-down”) by NMR (207) and high-resolution MS techniques, such as tandem MS (208, 209) and ion mobility MS (210). Sequencing intact GAG chains is still under development, and new technologies, such as solid-state nanopores (211, 212), are also being tested for this application.

New genomic technologies have been described to help link gene expression to functional changes in glycan structure at the cell surface. Single-cell RNA sequencing and spatially resolved transcriptomics have revolutionized our understanding of the positional context of gene expression in tissues (213). New tools were recently developed for detailed glycan analysis at the single-cell level (214, 215). These tools will enhance our ability to measure glycan diversity across cell types and understand their dysregulation in human disorders. One could imagine combining spatial transcriptomics with new mass spectrometry imaging techniques (216) to obtain detailed GAG profiles in tissues. Innovations in the field of epigenetics have allowed genome-wide profiling of the localization and function of transcription factors, chromatin remodeling complexes, and chromatin status in diverse cell types and pathophysiological conditions. The role of epigenetics in controlling GAG assembly is still unclear (106), despite obvious applications for these tools to understand the genetic factors controlling the dynamic process of glycosylation. Certainly, understanding the regulation of GAG assembly would provide avenues for controlling their synthesis in different biological contexts, exploiting novel therapeutic targets for treating human disorders, and developing bioengineered-defined structures for GAG-based therapeutics.

## GRANTS

Funding provided by University of Georgia Research Foundation (to R. J. Weiss) and NIH T32 training Grant GM107004 (to N. G. Patel).

## DISCLOSURES

No conflicts of interest, financial or otherwise, are declared by the authors.

This article is part of the special collection “Deciphering the Role of Proteoglycans and Glycosaminoglycans in Health and Disease.“ Liliana Schaefer, MD, served as Guest Editor of this collection.

## AUTHOR CONTRIBUTIONS

R.J.W. conceived and designed research; E.D.N. and R.J.W. analyzed data; A.B., N.G.P., and R.J.W. prepared figures; A.B., N.G.P., and R.J.W. drafted manuscript; A.B., N.G.P., E.D.N., and R.J.W. edited and revised manuscript; A.B., N.G.P., E.D.N., and R.J.W. approved final version of manuscript.
